# Exosome-mediated post-transcriptional oncogene regulation as a novel cancer therapeutic strategy

**DOI:** 10.1007/s12672-026-05255-y

**Published:** 2026-05-23

**Authors:** Bunty Sharma, Kawaljit Singh Kaura, Ranjay Kumar Choudhary, Suresh Babu Kondaveeti, Malathi Hanumanthayya, Abhishek Chauhan, Sanjana Gupta, Himanshu Sharma, Anuj Ranjan, Ambrish Mishra, Ujjawal Sharma

**Affiliations:** 1 Department of Biochemistry, All India Institute of Medical Sciences, Bathinda, 151001 India; 2 Department of Urology, All India Institute of Medical Sciences, Bathinda, 151001 India; 3 College of Applied and Health Sciences, A’Sharqiyah University, P.O. Box 42, 400 Ibra, Sultanate of Oman; 4 Department of Biochemistry, Symbiosis Medical College for Women, Symbiosis International (Deemed University), Pune, India; 5 Department of Biotechnology and Genetics, School of Sciences, JAIN (Deemed to be University), Bangalore, Karnataka India; 6 Amity Institute of Environmental Toxicology, Safety and Management, Amity University, Noida, Uttar Pradesh India; 7 Department of Biotechnology, Himachal Pradesh University, Shimla, Himachal Pradesh India; 8 Department of Human Genetics and Molecular Medicine, Central University of Punjab, Bhatinda, 151001 India

**Keywords:** Exosomes, Post-transcriptional regulation, Oncogene modulation, Tumor microenvironment, miRNA, lncRNA

## Abstract

Exosomes are tiny vesicles (30–150 nm in size) secreted by nearly every cell type that have lately emerged as essential regulators of intercellular communication and gene expression in cancer. They accommodate bioactive cargos such as miRNAs, lncRNAs, circRNAs, and mRNAs, all of which direct oncogene expression at the post-transcriptional level. Exosomal RNAs influence post-transcriptional and epigenetic regulatory mechanisms implicated in tumor activity, including mRNA degradation, translation repression and activation, alternative splicing interference, and epigenetic remodeling, which contribute to tumorigenic processes such as proliferation, angiogenesis, metastasis, immune evasion, and drug resistance. Tumor-derived exosomes also regulate the key oncogenic pathways such as PI3K/AKT, JAK/STAT, and Wnt/β-catenin to promote tumor stroma remodeling, thereby inducing macrophage M2 polarization, fibroblast transformation into cancer-associated fibroblasts, and pre-metastatic niche formation, favoring metastases. Targeting exosome-mediated oncogenic communication has therapeutic potential. Strategies include inhibiting exosome biogenesis and release using GW4869 or blocking Rab GTPases, blocking exosome uptake, and modulating oncogenic RNA cargo using antisense oligonucleotides, RNA interference, or CRISPR/Cas13-mediated RNA editing. Engineered exosomes also serve as natural, biocompatible carriers for the therapeutic delivery of siRNAs, miRNA mimics, mRNAs, or CRISPR components, offering improved stability, specificity, and reduced immunogenicity compared to synthetic counterparts. There are significant translational challenges, including large-scale manufacturing, purification, standardization, and biosafety testing, despite promising preclinical and early clinical results. In summary, comprehending and implementing post-transcriptional oncogene regulation via exosomes is a transformative strategy in precision oncology, creating new opportunities in targeted diagnosis, prognostication, and advanced cancer therapies.

## Introduction

The synthesis of the protein product requires several steps once a gene has been transcribed. mRNA processing, nucleo-cytoplasmic export, mRNA localization, mRNA stability, and translational regulation are the components of these post-transcriptional events. As our understanding of particular post-transcriptional systems grows, it becomes increasingly clear that abnormalities in these pathways are connected to disease processes like cancer [1].

Numerous cell types, including lymphocytes, dendritic cells (DCs), and tumor cells, release exosomes, which are tiny membrane vesicles with diameters ranging from 30 to 150 nm. Exosomes, which are secreted by tumors, serve as effective messengers in cell-to-cell communication and play a crucial role in controlling the aggressiveness of tumors. Proteins, mRNAs, and microRNAs (miRNAs) found in tumor-derived exosomes can be transported between various cell types and even to distant locations to affect the biological activities of tumors, including stimulation of angiogenesis, invasion and metastasis, immunoregulation, premetastatic niche formation, and proliferation [2]. Post-transcriptional regulation of oncogenes primarily occurs within single cells, with miRNAs, long non-coding RNAs (lncRNAs), and RNA-binding proteins influencing mRNA stability and translation in a cell-specific manner. Exosome-mediated post-transcriptional regulation of oncogenes provides a unique intercellular regulatory mechanism, enabling the horizontal transfer of regulatory RNAs between tumor cells and from the tumor microenvironment. Exosomes utilize selective RNA cargo loading and targeted delivery to spatially and temporally separate RNA biogenesis from RNA function, enabling oncogenic regulatory signals produced in tumor cells to reprogram recipient cells. This regulatory mode enables coordinated tumor progression, immune evasion, and the formation of metastatic niches, thereby extending post-transcriptional control beyond intracellular limits and introducing a more complex layer of gene regulation that traditional mechanisms cannot achieve alone [3–5]. The most extensively studied functional payloads in exosomes are miRNAs. Target genes’ 3’ untranslated regions are bound by miRNAs, which are small RNAs (21–23 nucleotides) that quickly degrade the target transcript and suppress translation [6, 7]. Through various pathways and mechanisms, many miRNAs contribute to the development of cancer. According to Jiang et al., two miRNAs (miR-9 and miR-181a) extracted from tumor exosomes can trigger the JAK/STAT signaling pathway and promote the growth of early-stage myeloid-derived suppressor cells in breast cancer, resulting in immunological escape and tumor progression [8].

Exosomes generated from Merkel cell cancer exhibit elevated expression of miR-375. The miR-375, which is produced by cancer cells, enters the fibroblasts and functions as a shuttle miRNA. Through the p53 pathway, the fibroblasts undergo polarization and transform into cancer-associated fibroblasts (CAFs). This produces an environment that is pro-tumorigenic and conducive to the growth of cancer. Additionally, exosomal miRNAs serve as intermediaries between distant organs and the primary tumor cells. To create the pre-metastatic niche and encourage tumor metastasis, effective communication is essential [9, 10]. It has been demonstrated that exosomes and macrovesicles secrete both conventional Wnt ligands, such as Wnt3a, and noncanonical Wnts, like Wnt5a. Furthermore, it has been demonstrated that Wnt-carrying exosomes can function as signaling messengers in a variety of cancers, including colorectal, pancreatic, lung adenocarcinoma, breast, and diffuse large B-cell lymphoma [11]. According to a recent study, the Wnt-receptor Fzd10 can also sustain and reestablish the growth of colorectal, gastric, hepatic, and bile duct cancer cells, as it is transported through exosomes. Viability was restored when FZD10-silenced cells were treated with exosomes of non-silenced cells; the Fzd10 protein and mRNA levels suggest that FZD10 plays a role in long-distance metastasis and cancer reactivation [12]. Moreover, extracellular vesicles (EVs) have been discovered to transport intracellular elements of the Wnt signaling pathways, including β-catenin [13].

The oncogenic composition of tumor-derived exosomes differs from that of exosomes generated by healthy normal cells, and the oncogenic content varies further among cancer cells. Additionally, it has been demonstrated that responses to anticancer therapy and tumor formation are correlated with oncogenic content. Tumor-derived exosomes typically carry elements that encourage cancer cells to proliferate, invade, and acquire medication resistance. Thus, these tumor-derived exosomes play a crucial role in the interactions between cancer cells and the tumor microenvironment. Through the transmission of signaling molecules and regulatory biomolecules, exosomes generated by non-cancerous cells within the tumor microenvironment actively interact with cancer cells, affecting tumor behavior. Important processes, such as tumor development, metastasis, immune evasion, and treatment resistance, are all regulated by this bidirectional interaction [14–16].

Exosomes have been explored as tumor biomarkers, therapeutic targets, and drug delivery systems for tumor detection, prognosis, and treatment due to their role in intercellular communication. Notwithstanding the numerous benefits of exosomes, their production and utilization present several challenges, including a lack of comprehensive understanding of biological processes and concerns regarding safety and specificity for therapeutic purposes [17]. Lastly, using exosomes to carry RNA therapies or antisense oligonucleotides has the potential to specifically alter the harmful post-transcriptional regulatory circuits of cancer.

In order to highlight exosome-mediated oncogene regulation as a novel and practical cancer therapeutic approach, this review is organized around a single framework that includes exosomal cargo classification, post-transcriptional regulatory mechanisms, tumor microenvironment crosstalk, therapeutic strategies, and clinical translation.

## Exosomal cargo and oncogene regulation

**Fig. 1:**
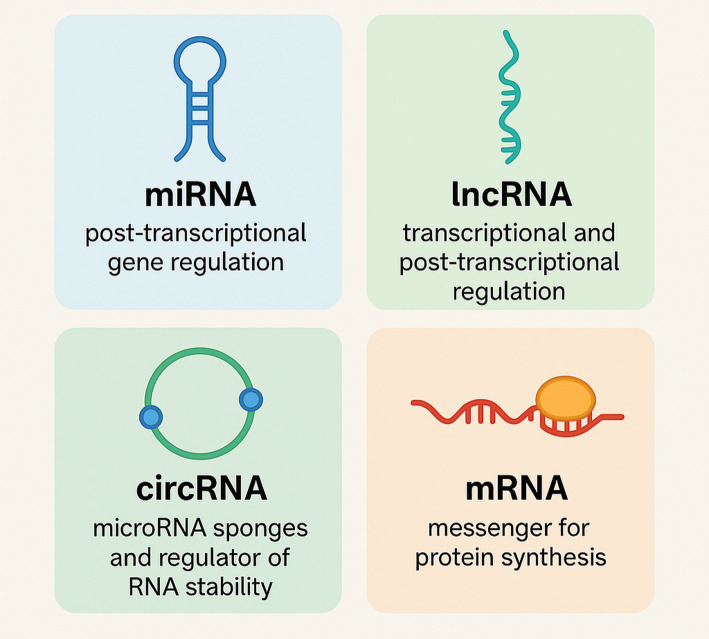
Exosomal RNAs includes both ncRNAs(miRNA, lncRNA, circRNA) and coding RNA(mRNA)—mediate gene regulation and intercellular communication

Exosomes are essential components of the tumour microenvironment that facilitate intercellular communication by transporting a variety of molecules, like non-coding RNAs (ncRNAs), including circular RNAs (circRNAs), lncRNAs, and miRNAs and coding RNAs (mRNA) (Fig. 1). Exosomes preferentially integrate these ncRNAs, which are endowed with regulatory roles, and these ncRNAs are also involved in important cancer-causing activities, including tumour development, spreading, metastasis, angiogenesis, treatment resistance, and avoiding the immune system [18, 19]. It was first discovered in sheep reticulocytes in 1983. Exosomes are tiny EVs that range in width from 30 to 150 nm. They are mostly produced from multivesicular bodies (MVBs) [20]. Exosomes are intricate structures mostly made up of proteins, lipids, and nucleic acids. A lipid bilayer, mostly composed of phosphatidylcholine, phosphatidylethanolamine, phosphatidylinositol, phosphatidylserine, and sphingomyelin, makes up the exterior structure. These lipid molecules content and composition significantly determine the stability and functional characteristics of exosomes [21]. Exosomes membranes may include proteins or be located in phospholipid domains known as lipid rafts. These proteins fall into two categories: non-specific proteins, which are present in all exosomal types and are frequently used as exocytosis markers (e.g., CD9, CD63, CD81, and HSP70), and specific proteins, which differ based on the parent cell’s origin and include markers like MHC II in B-lymphocyte exosomes [22] and perforins and granzymes in T-lymphocyte exosomes [23].

### Types of RNA found in exosomes: miRNAs, lncRNAs, circRNAs, and mRNAs

#### miRNAs

miRNAs, a category of short ncRNAs approximately 22 nucleotides in length, play a pivotal role in controlling gene expression at the post-transcriptional level [24]. The nucleus is where miRNA manufacturing begins, where DNA is converted into primordial miRNAs, or pri-miRNAs, which can have a length of several thousand bases. A protein called RNA polymerase II facilitates this transcription. Then, the DGCR8-Drosha complex converts the pri-miRNAs into precursor miRNAs, or pre-miRNAs. Exportin 5, a transport protein, exports these pre-miRNAs from the nucleus. The enzyme Dicer further breaks down pre-miRNAs in the cytoplasm to produce single-stranded miRNAs. After that, mature miRNAs are sorted into exosomes using various methods [25]. In the case of miRNAs, the role of RNA binding proteins (RBPs) in RNA loading into EVs has been more thoroughly described. A number of RBPs have been demonstrated to interact with particular EV-enriched miRNAs, including hnRNPA2B1, hnRNPA1, and hnRNPC. This interaction may affect miRNA sorting into EVs [26]. The sorting of miR-223 into exosomes is mediated by YBX1-forming cytosolic condensates, another RBP. Furthermore, EV miRNA sorting in inflammatory conditions is linked to the RBP FMR1. It’s worth noting that certain non-RBP proteins may also help miRNAs sort into EVs. For example, connexin 43 may mediate the loading of certain miRNAs into HEK293 EVs by interacting with a subset of miRNAs that have stable secondary structure elements, such as double-stranded hairpin loops, like miR-133b [26–28].

Several studies have demonstrated that components of the RNA-induced silencing complex, particularly Argonaute 2 (AGO2), play a crucial role in the miRNA loading process. Furthermore, the RNA-binding protein hnRNPA2B1 is known to identify specific miRNA EXO-motifs and facilitates the selective packaging of miRNAs in a manner dependent on sumoylation. In contrast, YBX1 has been shown to regulate the sorting of specific subsets of miRNAs, independent of endosomal sorting complexes needed for transport (ESCRT) components. This underscores the existence of multiple concurrent pathways that contribute to the enrichment of miRNAs in exosomes [29–32]. Post-transcriptional gene silencing, which is facilitated by the miRNA-induced silencing complex, is the primary gene regulatory function of miRNAs. Argonaute 2 (Ago 2), the trinucleotide repeat-containing gene 6 (TNRC6), and miRNAs mediate three essential stages in the traditional miRISC process: (1) Ago2 attracts TNRC6, which in turn attracts the CCR4-NOT deadenylase complex, causing mRNA to deadenylate and degrade. (2) TNRC6 recruits the DCP1/2 decapitation complex and cleaves the 5’ cap of mRNA, decreasing mRNA stability. (3) When Ago 2 binds, the mRNA can’t attach to the ribosome, which prevents translation. MiRNAs play a crucial role in cancer development and are commonly dysregulated in malignant tumors [33]. Tumour types differ in this dysregulation; however, miRNAs, including miR-221, miR-222, miR-146b, and miR-155, are often reported to be abnormal [34]. In particular, gastrointestinal, hepatocellular, and papillary thyroid malignancies exhibit overexpression of miR-221 and miR-222, which target and inhibit tumour suppressor genes [35].

#### lncRNAs

lncRNAs, characterized as transcripts longer than 500 nucleotides, are predominantly synthesized by RNA polymerase II. Many lncRNAs undergo typical RNA processing activities, including splicing and polyadenylation, leading to their categorization as “mRNA-like” transcripts. Growing data suggest that lncRNAs display structural and biogenetic diversity. A notable subset of lncRNAs lacks poly(A) tails or 7-methylguanosine caps, with certain lncRNAs transcribed from RNA polymerase I or III promoters, including those linked to ribosomal RNA loci. Furthermore, numerous lncRNAs arise from noncanonical processing mechanisms, such as cleavage from intronic regions or repetitive genomic sequences, underscoring the varied origins and regulatory intricacies of this RNA family [36]. Numerous lncRNAs are exported to the cytoplasm and have been identified in human blood exosomes; however, the processes that control their sorting are still unknown, although it is likely to involve interactions between specific lncRNA sequences and RNA-binding proteins [37]. The sorting of lncRNAs into exosomes seems to be primarily dependent on their interactions with RBPs, rather than being governed by specific sequence motifs. Proteins such as YBX1, HuR (ELAVL1), and hnRNPK are linked to the selective export of carcinogenic lncRNAs, including HOTAIR and MALAT1, particularly in stressful or oncogenic conditions. The findings suggest that the sorting of lncRNA is influenced by the creation of RNA–protein complexes, particular subcellular localization, and the overall cellular environment, rather than according to a standardized set of processing principles [38–42]. The hnRNPA2B1 binds to particular motifs in long RNAs (both mRNA and lncRNA) and mediates their sorting into EVs from endothelial cells. However, the mechanisms of long RNA loading into EVs are not as well understood as those of miRNA. GGAG-, UAG-, or GC-contained motifs, which are recognized by classical RBPs like RBM5, LIN28A, RBM28, and hnRNPA2B1, are abundant in the identified sequences, indicating their possible role in loading these RNAs into EVs [43].

Exosomal lncRNAs are important discriminant indicators for various disorders, including cancer, due to their highly specific expression patterns. Although there has been minimal overlap in reports of very similar findings in a particular cancer, exosomal lncRNA expression differs in different types of cancer and at different stages of a particular cancer, making lncRNAs essential indicators for cancer diagnosis and prognosis [44]. By delivering their molecular cargoes to recipient cells, EVs facilitate cell-to-cell communication. EV-mediated distribution of lncRNA has been shown to affect various functions, including immune response, chemosensitivity, and tumor growth and development, according to accumulating data. The adjacent counterparts in the tumor microenvironment acquire aggressive and chemo-resistant characteristics due to tumor-derived EVs-lncRNA. In the meantime, EVs-lncRNA modifies the surrounding environment to promote tumor development and progression via mediating the interaction between tumor and stromal cells [45]. The diverse actions of exosomal miRNAs are mirrored by exosomal lncRNAs, which are crucial elements of the tumour microenvironment and play significant roles in cancer development. Among other things, these functions include fostering angiogenesis, immunological evasion, tumour growth, metastasis, and treatment resistance [41, 42, 46]. Exosomal lncRNAs such as HOTAIR, UCA1, and MALAT1 contribute to tumor progression, angiogenesis, and therapy resistance through modulation of gene expression in recipient cells within the tumor microenvironment [38, 39, 47].

#### circRNAs

Due to their stable, closed-loop shape and involvement in various biological functions, circRNAs, a distinct family of ncRNAs, have garnered attention. They are highly attractive prospects for therapeutic applications due to their exceptional stability and abundance in biological fluids, particularly when encapsulated within exosomes [48]. A type of ncRNA known as circRNA is distinguished by its distinct closed-loop structure. In contrast to traditional linear splicing, they are created via reverse splicing, which joins the upstream 3’ splice site with the downstream 5’ splice site in reverse order [49]. circRNAs demonstrate significant enrichment in exosomes compared to their linear equivalents. This characteristic is mostly due to their covalently closed circular structure, which provides enhanced stability. Experimental evidence indicates that the sorting of circRNAs is influenced by several parameters, including RNA length, secondary structure, and interactions with certain RBPs. Nevertheless, the precise molecular signals governing the selection of circRNAs remain inadequately understood [50, 51]. Researchers have shown that circRNAs in exosomes, such as circHIPK3 and circRNA_100284, can influence the development and death of cancer cells by acting as miRNA sponges and altering subsequent signalling pathways [52]. RBPs can be trapped in the cytoplasm by circRNAs, which prevent them from entering the nucleus and regulate their function. CircRNAs have the ability to bind to RBPs and alter their location or function, which has a substantial impact on gene regulation and cancer development [53]. For instance, circRNA_100338 influences RNA processing and metastasis in hepatocellular carcinoma (HCC) by interacting with the splicing regulator NOVA2. Through these interactions, circRNAs can function as scaffolds, attracting RBPs to specific transcripts or blocking their nuclear entry, thereby influencing the behavior of cancer cells [54]. Exosomal circRNAs, such as CircSATB2, are prevalent in non-small cell lung cancer (NSCLC) cells and can be transferred to other cells via exosomes, promoting cell proliferation, migration, and invasion in NSCLC. Additionally, circRNA-002178 transported by exosomes can be transferred to CD8 + T cells, thereby enhancing the production of programmed death-ligand 1/programmed cell death protein 1 in NSCLC [38, 39].

Because of their stable structure, they are promising candidates for biomarkers in liquid biopsies that can aid in early disease detection.

The process by which circRNA enters exosomes is of great research importance because of the various biological roles of exosomal circRNA that are presently being identified and validated. CircRNAs enter exosomes through the following mechanisms: (A) RBP-mediated, (B) miRNA-mediated, (C) RNA modification-mediated, and (D) mechanisms under cellular stress. By interacting with SNF8, a component of the ESCRT-II complexes, through its unique components, circRHOBTB3 can be selectively sorted into exosomes through an RNA-binding protein-mediated mechanism. hnRNPA2B can package circCCAR1, circ-CDYL, and circNEIL3 into exosomes. In a miRNA-mediated mechanism, miR-671-AGO2 mediates the degradation of circCDR1as in source cells, while exosomes containing miR-7 mimics can efficiently reduce the levels of competing endogenous spongy circCDR1as. RNA modifications may have an impact on the structure and function of circRNAs in RNA modification-mediated mechanisms. Circ-CDYL sorting into exosomes can be facilitated by the M6A modification of circ-CDYL [51, 55].

#### mRNAs

Exosomes also transport fragmented and full-length mRNA transcripts, which can be translated into proteins in destination cells. Evidence suggests that mRNA sorting is partially facilitated by RBP–mRNA complexes, including proteins such as YBX1 and HuR, which recognize specific sequence or structural characteristics and promote vesicular trafficking. Moreover, elements of ESCRT and lipid-raft-associated pathways facilitate mRNA accumulation within ILVs. During ILV production in MVBs, RNA is packaged into exosomes. This process involves the selective recruitment of mRNA fragments to certain parts of the MVB limiting membrane that have lipid microdomains with ceramide, cholesterol, and sphingolipids. Certain sequence motifs are recognized by RBPs such as hnRNPA2B1, which then direct RNAs to specific locations. Lipid modifications make binding stronger. Ceramide synthesis helps membranes bend and bud, making it easier for RNA to enter ILVs without ESCRT, instead using lipids. As a result, many exosomes contain very little RNA due to selective binding, with specific transcripts included based on their sequence and membrane affinity [56]. Stressors such as hypoxia or chemotherapy modify the mRNA cargo profile of exosomes, thereby enhancing the export of transcripts related to proliferation, angiogenesis, and therapeutic resistance [57, 58]. Due to their extensive roles in tumor growth and modification of the microenvironment, the mRNAs carried by EVs in cancer have recently garnered considerable attention. mRNA molecules are a promising diagnostic tool for cancer because they are encapsulated within the lipid bilayer membrane, which keeps them stable in the bloodstream. Furthermore, EVs can be employed as biological therapies in both their original and modified forms. EV-carried mRNA can be translated into proteins in target cells, resulting in a variety of advantageous phenotypes for cancer cells. We anticipate that EV-derived mRNAs carry out numerous other crucial roles in cancer pathogenesis, given the functions of mRNAs in EVs that are already known, such as the modulation of tumorigenesis and the tumor microenvironment by the promotion of angiogenesis, the immune response, drug resistance, and invasion, and the remodeling at distant sites that induces metastasis [59]. The transfer of mRNAs via exosomes provides a novel mechanism of horizontal gene transfer, contributing to phenotypic changes in target cells [4].

For instance, glioblastoma cells send out exosomes that contain EGFRvIII mRNA. This mRNA can then move to endothelial cells and help tumours grow new blood vessels [60]. Additionally, utilizing exosomes to deliver mRNA could be beneficial for gene therapy and regenerative medicine.

#### Synergistic regulatory effects of different RNA types

While miRNAs, lncRNAs, circRNAs, and mRNAs are often discussed separately, there is mounting evidence that multiple RNA types within exosomes function as interconnected regulatory networks in recipient cells. Different RNA cargos can be delivered by exosomes simultaneously, allowing for layered translational control, RNA–protein cooperation, and competing endogenous RNA (ceRNA) interactions that alter oncogene expression in various cells.CircRNAs and lncRNAs functioning as endogenous “miRNA sponges” is a well-known mechanism of synergy. Exosomal circRNAs like circRNA_100284 have been demonstrated to bind and sequester tumor-suppressive miRNAs (like miR-217), increasing the expression of downstream oncogenic targets like EZH2 and cyclin D1 and stimulating recipient cell proliferation. Exosomal lncRNAs can also sponge miRNAs to alter regulatory networks. In cancer models, for instance, LNCARSR and lncRNA-ATB have been shown to sequester specific miRNAs, thereby improving migration and chemoresistance [61–63].

RNA–protein complexes carried by exosomes provide an additional level of control, in addition to ceRNA interactions. Some exosomal circRNAs, such as circRNA-SORE, bind to RBPs, including YBX1, to prevent their degradation and influence downstream signaling pathways associated with drug resistance. After being taken up by recipient cells, RBPs like hnRNPA2B1 and YBX1 actively sort and stabilize different RNAs within exosomes to form functional complexes that can alter post-transcriptional gene regulation [58, 64, 65].

### Mechanisms by which these RNAs regulate oncogene expression

In cancer biology, exosomal RNAs—particularly ncRNAslike, circRNAs, lncRNAs, and miRNAs and coding RNAs like mRNAs—are increasingly significant post-transcriptional regulators. They affect key biological mechanisms, such as translational control, alternative RNA splicing, and mRNA stability and degradation, which alter the way recipient cells produce oncogenes. Thus, cells that receive oncogenes exhibit altered patterns of gene expression. These EVs, which contain these RNAs, form a strong communication channel within the tumour microenvironment that facilitates horizontal gene transfer between cells and often leads to the acquisition of pro-tumourigenic traits in healthy cells [3, 66].

#### Regulation of mRNA stability and degradation

Modifying mRNA stability is one of the most straightforward ways exosomal RNAs influence oncogene expression. Partial sequence complementarity enables exosomal miRNAs to bind to the 3′ untranslated regions (3′ UTRs) of target tumor-suppressor or oncogenic mRNAs, potentially causing the mRNA to be degraded or rendered unstable. This mechanism is generally handled by the RNA-induced silencing complex (RISC), which either suppresses poly(A)-tail extension or guides mRNA cleavage. For example, exosomal miR-21, which is routinely produced by cancer cells, targets tumour suppressors such as PTEN and PDCD4. By post-transcriptionally inhibiting important tumor suppressors involved in PI3K/AKT regulation, exosomal miR-21 increases oncogenic signaling. After being taken up by recipient cells, miR-21 enters the RISC and binds to the 3 ′ untranslated regions of PTEN and PDCD4 mRNAs, promoting either translational repression or degradation of these mRNAs. Increased intracellular PIP3 levels resulting from PTEN loss led to prolonged AKT activation and enhanced cell survival, proliferation, and metabolism. Tumor growth and treatment resistance are caused by the simultaneous suppression of PDCD4, which also releases inhibitory control over oncogenic pathways [67]. Through the PI3K/AKT pathway, this leads to enhanced oncogenic signaling and reduced expression [67]. This selective degradation of mRNAs ensures that tumour-promoting genes are preferentially translated, whereas tumour suppressors are downregulated.

#### Translational repression or activation

miRNAs and lncRNAs, among the exosomal RNAs, were the most well-studied exosomal ncRNAs in terms of post-transcriptional gene regulation, which exerts regulatory effects in terms of mRNA translation in recipient cells. They bind to the 3’ UTRs of target mRNAs mainly through partial nucleotide sequence homology and usually repress their translation or induce their degradation by recruiting them to RISC [68]. This repression may be reversible and does not necessarily lead to mRNA decay, which implies that the mechanism controlling protein synthesis in response to the cellular environment is dynamic.

**Fig. 2:**
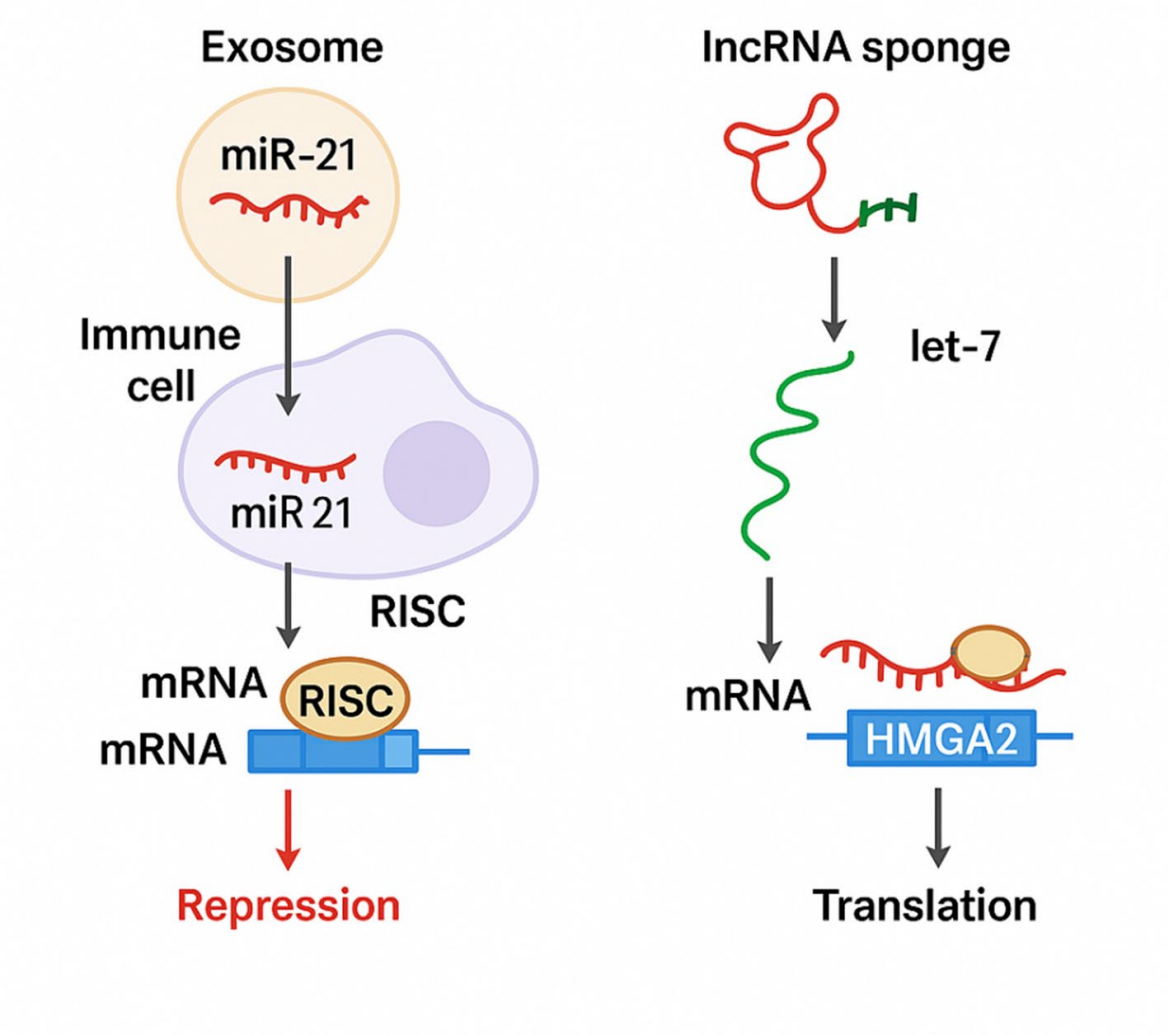
Exosomal ncRNA-Mediated Translational Control. Exosomal miR-21 delivered into immune cells binds mRNA via the RISC complex, leading to translational repression (left). In contrast, lncRNA acts as a sponge for let-7 miRNA, preventing its binding to HMGA2 mRNA and thereby promoting translation (right)

Exosomal miR-21 originating from the tumor can be internalized by the immune cells of recipients, downregulating PDCD4 and PTEN expression and thus promoting tumor immune evasion and progression at the post-transcriptional level [69]. A notable example is lncRNA H19, which, when packaged in exosomes from colorectal cancer cells, binds members of the let-7 family of RNA—a tumor-reducing mRNA group. By sponging let-7, exosomal H19 gains control over HMGA2, an oncogene known to promote the growth and metastasis of tumors (Fig. 2) [70].

#### RNA splicing modulation

**Fig. 3:**
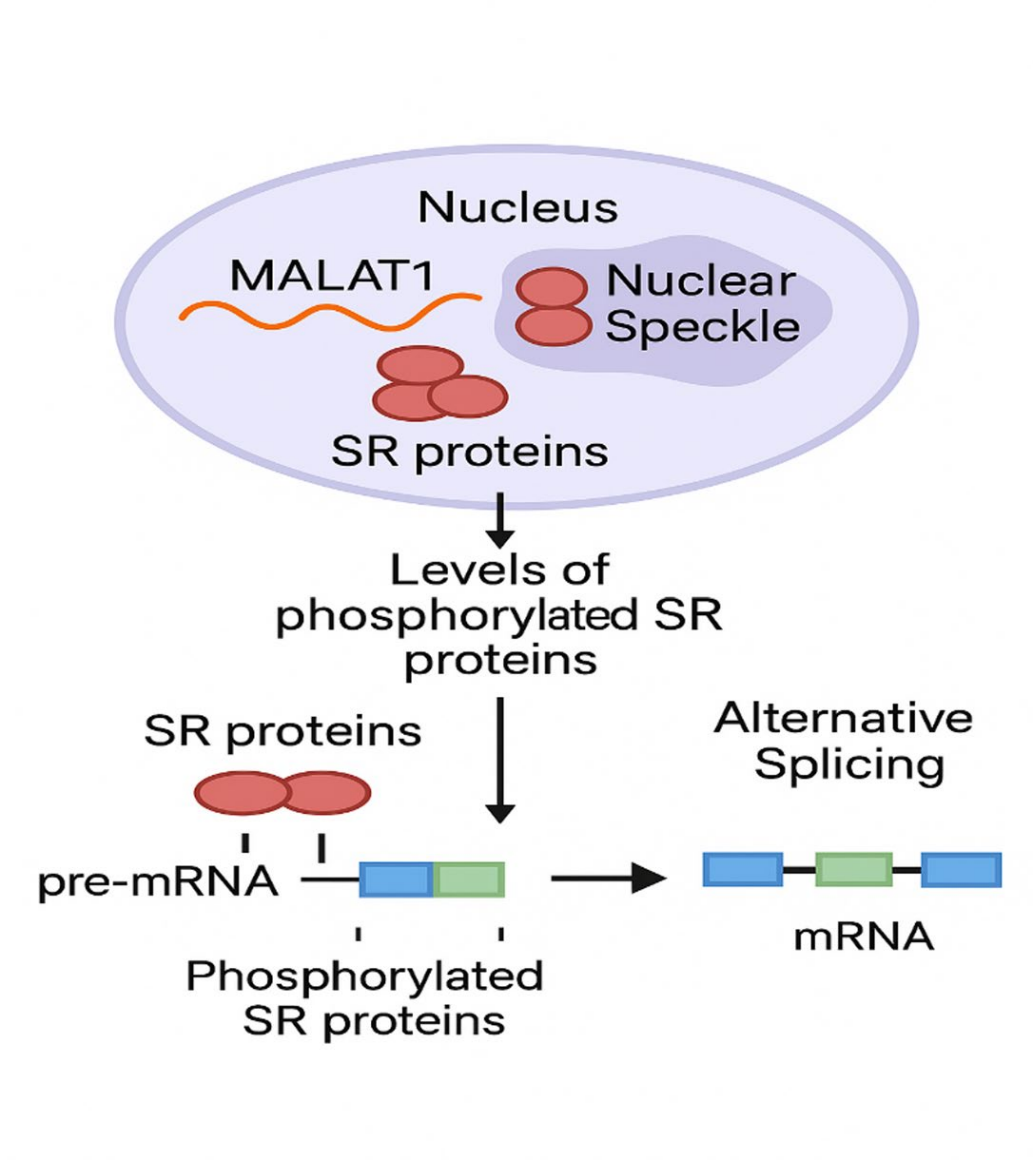
MALAT1-mediated regulation of alternative splicing via modulation of SR protein phosphorylation within nuclear speckles, impacting exon inclusion during pre-mRNA processing

Higher eukaryotes use pre-mRNA alternative splicing (AS) to expand the complexity of their transcriptome and proteome. The serine/arginine (SR) splicing factors regulate AS that is tissue- or cell-type-specific in a manner that is dependent on both phosphorylation and concentration. It is yet unknown what processes control the amounts of active SR proteins in cells. It shows that the long nuclear-retained regulatory RNA, MALAT1, plays a part in the control of AS. MALAT1 affects the distribution of this and other splicing factors in nuclear speckle domains via interacting with SR proteins. The AS of a comparable group of endogenous pre-mRNAs is altered by MALAT1 depletion or by overexpression of an SR protein (Fig. 3). Additionally, MALAT1 controls the amounts of phosphorylated SR proteins in cells [71].

## Tumor microenvironment and exosomal influence

Tumor progression is increasingly recognized as a dynamic interplay between cancer cells and their surrounding microenvironment, where exosomes—nano-sized EVs—act as vital mediators of intercellular communication. These vesicles carry a wide range of bioactive cargo, including proteins, lipids, and various RNA species, and serve as shuttles that influence the behaviour of neighbouring and distant cells. Fatima and Nawaz, [72] emphasized that stem cell-derived exosomes maintain cellular stemness and also promote the transformation of fibroblasts into tumor-supportive cells, thus participating in stromal remodeling and the generation of a permissive tumor niche. Exosomes facilitate immune evasion by modulating the phenotypes of immune cells, particularly tumor-associated macrophages (TAMs). The polarization of macrophages from the pro-inflammatory M1 phenotype to the tumor-promoting M2 subtype is strongly influenced by exosomal cargo.

### Roles in proliferation and invasion

Multiple studies have shown that tumor-derived exosomes induce M2 polarization through diverse molecular mechanisms. For example, exosomes carrying miR-301a-3p derived from hypoxic pancreatic cancer cells promote M2 polarization via PTEN suppression and PI3Kγ pathway activation, leading to enhanced epithelial-mesenchymal transition (EMT), invasion, and metastasis [73] (Table 1).

### Metastasis and angiogenesis

Tumor-derived exosomes not only modulate immune cells but also facilitate communication with other cells, such as tumor-associated endothelial cells and mesenchymal stem cells (MSCs). This interaction is crucial as it leads to the reconfiguration of the tumor microenvironment, ultimately fostering processes such as angiogenesis, invasion, and metastasis. Tumor exosomes modify the behavior of endothelial cells to support both angiogenesis and lymphangiogenesis. According to Arcucci et al. [74], ncRNAscarried in exosomes, such as miRNAs and lncRNAs, reprogram endothelial gene expression, enhancing vascular permeability and sprouting. In colorectal cancer, miR-106b-5p in exosomes directly downregulates PDCD4 in macrophages and activates PI3Kγ/AKT/mTOR signaling, forming a feedback loop that reinforces EMT and metastasis [75]. Another aspect of exosome-mediated communication is their role in preparing distant organs for metastasis. Wortzel et al. [76] discussed how exosomes from primary tumors can “educate” the microenvironment of distant tissues, facilitating the formation of pre-metastatic niches. These niches are characterized by increased extracellular matrix remodeling, angiogenesis, and immune tolerance, all of which are conducive to future metastatic colonization. Increasing evidence suggests that exosomes originating from tumors contribute to the formation of organ-specific pre-metastatic niches, rather than inducing a generic systemic priming effect. The observed specificity is primarily due to the presence of exosomal surface molecules, particularly integrins, along with the lipid composition and the selective cargo of RNA and proteins. All of these things together affect how well resident cells in target organs take up substances. Hoshino et al., demonstrated that tumor exosomes exhibit distinctive integrin profiles, which promote organ-specific homing. Exosomes that have more integrins α6β4 and α6β1 tend to go to the lung tissue, while exosomes that have more integrin αvβ5 tend to go to the liver. In the lung, these exosomes interact with pre-existing fibroblasts, epithelial cells, and Kupffer cells. This activates Src signaling pathways and increases the production of the pro-inflammatory S100 gene. This process alters the extracellular matrix, increases blood vessel permeability, and facilitates inflammatory priming. Exosomal RNAs and proteins, such as miRNAs that activate the STAT3, TGF-β, and NF-κB signaling pathways, amplify these changes. These elements work together to create a microenvironment that is good for metastatic colonization. The regulatory mechanisms unique to specific organs underscore the importance of exosome-mediated intercellular communication in shaping metastatic patterns and in priming distant locations for tumor development [77]. Exosomal miRNAs and proteins, such as VEGF, miR-21, and miR-25-3p, play a crucial role in activating angiogenic signaling pathways, including PI3K/Akt and MAPK/ERK, in endothelial cells. These components significantly enhance cellular functions, including growth, movement, and tube formation. Exosomes from colorectal cancer that carry miR-25-3p have been found to preferentially target endothelial cells. This interaction causes VEGFR2 to be upregulated, which makes blood vessels more permeable. This process is a crucial component of forming the pre-metastatic niche [78].

### Immune remodeling

Similarly, exosomes from glioma cells enriched with miR-1246 target TERF2IP in macrophages, leading to STAT3 activation and NF-κB inhibition, which skew macrophages toward an M2 phenotype and suppress immune responses [79]. These findings demonstrate how tumor-derived exosomes rewire macrophage function to support immune suppression and tumor expansion. The phenomenon is not restricted to macrophages; exosomes also impact T-cell activation and can carry ligands such as PD-L1, leading to T-cell exhaustion and reduced cytotoxicity [80]. Tumor-derived exosomes transport several miRNAs that significantly suppress T-cell activation and promote the differentiation of regulatory T cells, thereby aiding immune evasion strategies. Exosomes originating from prostate cancer have been demonstrated to enhance the expression of CD73 on DCs, hence inhibiting antigen presentation and suppressing T-cell activation. Moreover, exosomal miRNAs derived from melanoma, including miR-3187-3p, miR-498, miR-122, miR-149, and miR-181a/b, have been shown to impair T-cell receptor signaling and attenuate TNF-α release in T cells, thus resulting in diminished cytotoxic responses [81, 82]. Exosomes produced from tumors that express or contain PD-L1 can directly suppress the function of CD8⁺ T-cells. This inhibition leads to a reduction in activation indicators and interferes with essential signaling pathways, including as NF-κB and ERK. Thus, this pathway facilitates tumor development by suppressing immune responses [83]. A recent study on tongue squamous cell carcinoma revealed that exosomes from tumors rich in the lncRNASNHG26 can inhibit the growth and activation of natural killer cells by utilizing the TGF-β1/Smad2 signaling pathway. This discovery elucidates a molecular link between the transport of exosomal lncRNA and the inhibition of innate immunity [84]. Beyond immune modulation, exosomes significantly contribute to the remodeling of the tumor stroma. Fatima and Nawaz, [72] reported that exosomes are capable of morphologically and functionally converting fibroblasts into tumor-initiating fibroblasts, thus facilitating cancer cell invasion and growth. Tumor-derived exosomes affect MSCs, causing them to transform into CAFs or other cell types that suppress the immune system. Exosomes from breast cancer that are high in TGF-β1 and miR-21 can change the way MSCs work. This reprogramming leads to the release of extracellular matrix components, growth factors, and immunomodulatory cytokines, all of which facilitate the tumor’s growth and create a supportive microenvironment [85, 86].

### Therapy resistance

Exosomes also influence drug resistance in cancer. As described by Kulkarni et al. [87], exosomal miRNAs can modulate genes associated with multi-drug resistance, such as those encoding drug efflux pumps, apoptosis regulators, and DNA repair enzymes. These miRNAs not only affect the donor cancer cells but also spread resistance traits horizontally to nearby sensitive cells, further complicating therapy. The versatility of exosomes extends into their use as therapeutic agents. Su et al. [88] demonstrated that modifying the miRNA content of pancreatic cancer-derived exosomes could successfully reprogram M2 macrophages into the tumoricidal M1 phenotype, suggesting the potential for exosome-based immunotherapy. Similarly, Gonzalez et al. [89] explored exosome derivatives for bio-informational reprogramming, emphasizing their utility in inducing apoptosis and reversing malignant phenotypes in tumor cells.

The identified interactions suggest that exosomes serve as intercellular messengers, facilitating the coordination of immune and stromal components during the remodeling of the tumor microenvironment. Interaction with endothelial cells is crucial for angiogenesis and vascular remodeling. All of these processes collaborate to facilitate tumor growth, invasion, and dissemination.

### Biomarkers

Exosomes are also emerging as non-invasive biomarkers due to their presence in body fluids and the specificity of their cargo. Maia et al. [90] highlighted the clinical value of exosome profiling for cancer diagnosis and monitoring treatment response. Exosomal contents, reflective of the tumor’s molecular landscape, can be accessed through liquid biopsy and may provide real-time insights into tumor dynamics. Finally, epigenetic regulation within exosomes adds further complexity to their function. RNA modifications such as N6-methyladenosine (m6A) play crucial roles in RNA stability and translation. Zhang et al. [91] emphasized that m6A-modified RNAs within exosomes contribute to tumor adaptation under hypoxia, metabolic reprogramming, and immune suppression. These modifications regulate gene expression at the post-transcriptional level, thus reinforcing the adaptability and resilience of tumor cells within a hostile microenvironment. An overview of mechanisms, including cancer types, exosomal cargo, targeted cell types, mechanistic pathways, and biological effects, is presented in Table 1.

## Therapeutic targeting strategies

Exosome-mediated oncogene regulation has gained significant importance in oncology. The approach includes disrupting the communication network between cancer cells, impairing their proliferation and metastasis. Therapeutic strategies aimed at exosome-mediated post-transcriptional oncogene regulation can be broadly viewed from two complementary perspectives. One approach considers exosomes as therapeutic targets, with the objective of inhibiting their biogenesis, secretion, or uptake to interrupt the transfer of oncogenic RNAs and other bioactive molecules between cancer cells and the tumor microenvironment. The second approach treats exosomes as therapeutic carriers, taking advantage of their natural stability, biocompatibility, and intrinsic cell-targeting ability to deliver therapeutic nucleic acids such as antisense oligonucleotides, siRNAs, or CRISPR/Cas components to tumor cells or associated stromal and immune cells. Together, these strategies provide a balanced framework for either suppressing pathological exosome-mediated communication or harnessing exosomes as efficient delivery platforms for cancer therapy.

### Exosome biogenesis inhibition

**Fig. 4:**
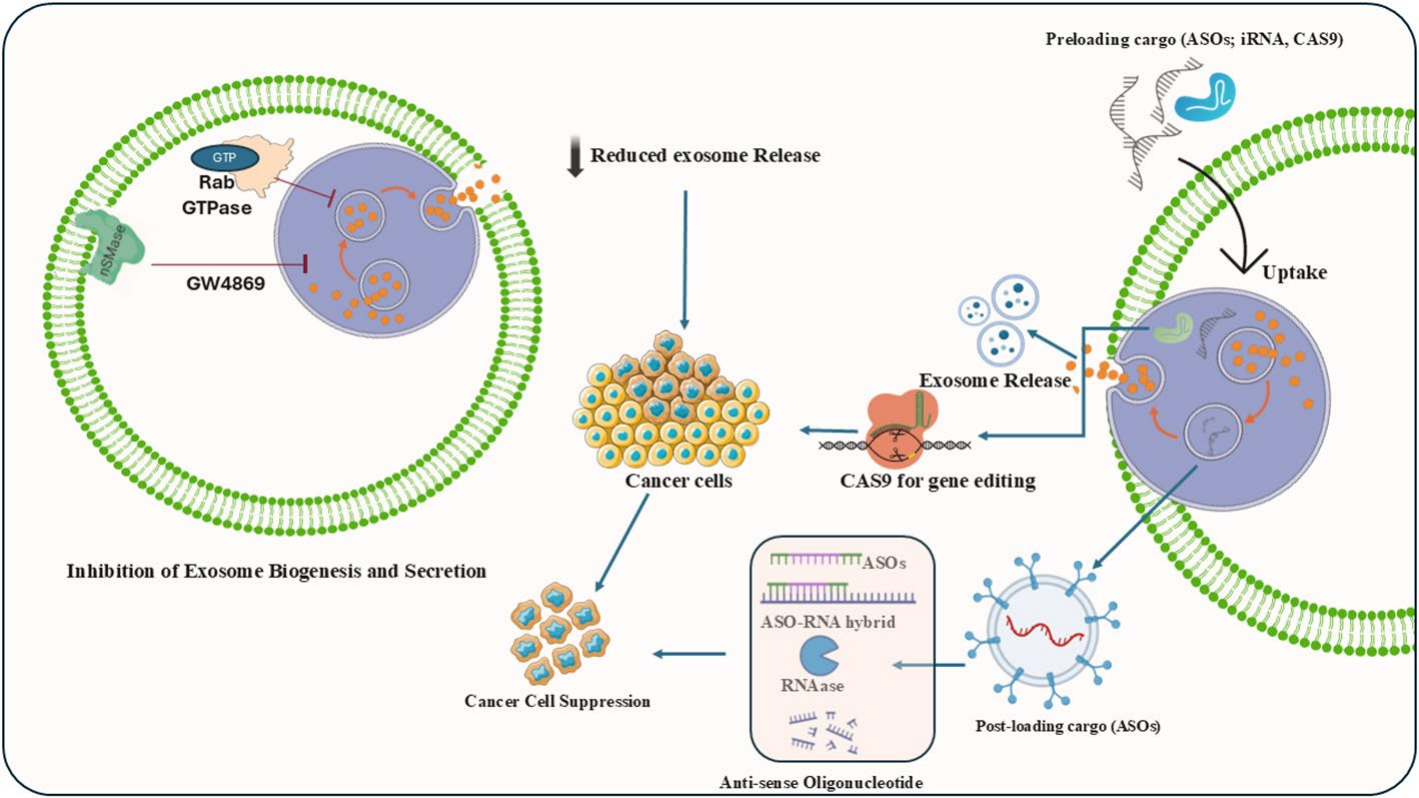
Schematic representation of strategies for inhibiting exosome biogenesis and secretion in cancer cells, including GW4869 treatment and Rab GTPase modulation, as well as therapeutic approaches using pre- and post-loading of cargos such as antisense oligonucleotides, RNAase, and CRISPR-Cas9 for targeted cancer cell suppression

Exosome biogenesis involves endosome formation, MVB formation, cargo sorting, and extracellular release, with mechanisms regulated by molecular pathways that are affected by its micro-environmental factors [92]. Various mechanisms mediating exosome biogenesis are believed to be favourable drug targets for cancer therapy, exhibiting potential benefits in treatment strategies. Dysregulation of post-transcriptional modifications can also impact the quantity and composition of exosomes, influencing cancer progression [93] (Fig. 4).

**Table 1:** Tumor-derived exosomes and their functional impact on the tumor microenvironment

Cancer type	Exosomal cargo	Target cell type	Mechanism of action	Biological outcome	References
Colorectal cancer	miR-106a-5p	Macrophages	SOCS6↓ → JAK2/STAT3↑	M2 polarization, liver metastasis	Liang et al. [94]
Colorectal cancer	miR-1246	Hepatic stellate cells	INSIG1↓ → SREBP2↑ → TLR4/NF-κB/TGF-β	HSC activation, metastasis	Liu et al. [83]
Glioma	miR-1246	Macrophages	TERF2IP↓ → STAT3↑, NF-κB↓	M2 polarization, immune suppression (inhibits CD8⁺ T-cell activation)	Qian et al. [79]
Pancreatic cancer	miR-155, miR-125b2 (engineered)	Macrophages (J774.A1)	Exosome cargo altered by nanoparticle transfection → shift from M2 to M1 phenotype	Reprogrammed macrophages to anti-tumor M1 type	Su et al. [88]
Pancreatic cancer	miR-301a-3p	Macrophages	PTEN↓ → PI3Kγ↑ → M2 polarization (hypoxia-induced)	Enhanced EMT, invasion, metastasis	Wang et al. [73]
Pancreatic cancer	FGD5-AS1 (lncRNA)	Macrophages (M2 TAMs)	p300 interaction → STAT3 acetylation → STAT3/NF-κB↑ → M2 polarization	Enhanced pancreativ cancer cell proliferation and metastasis	He et al. [95]
Colorectal cancer	miR-106b-5p	Macrophages	PDCD4↓ → PI3Kγ/AKT/mTOR↑ → M2 polarization	M2 polarization, EMT feedback loop, metastasis	Yang et al. [75]
Hepatocellular carcinoma	miR-628-5p	Tumor Cells (HCC)	METTL14↓ → m6A modification of circFUT8↓ → nuclear export↓ → tumor suppression	Suppressed HCC progression	Wang et al. [96]

#### Inhibition of neutral sphingomyelinase using GW4869

Neutral sphingomyelinase (nSMase) is a key enzyme that regulates ceramide production, which plays a crucial role in the formation of intraluminal vesicles (ILVs) within MVBs, leading to the release of exosomes. Inhibition of nSMase can reduce ceramide production, thereby impairing the inward budding of the endosomal membrane. This leads to decreased formation of ILVs within MVBs as a result, exosome biogenesis and secretion are significantly affected, thereby disrupting intercellular communication [97]. GW4869, a potent inhibitor of nSMase that has been shown to reduce cancer invasion by inhibiting the release of exosomes [98]. It can be used to sensitize cancer cells to chemotherapy drugs and reduce drug resistance in various types of cancer [99]. A study using GW4869 (20 µM) to inhibit exosome release in U937 acute myeloid leukemia cells resulted in a significant reduction in exosomal protein concentration and CD63-positive vesicles, confirming inhibited exosome secretion. The HPLC analysis confirmed that doxorubicin (PLD) was present in the exosomes, indicating that U937 cells exported the drug via exosomes as a resistance mechanism. Co-treatment with GW4869 + 0.5 µM PLD increased cytotoxicity to a level comparable to 1 µM PLD alone, as measured by both Annexin V/PI flow cytometry and LDH cytotoxicity assays (*P* < 0.05), suggesting GW4869 enhanced intracellular retention and efficacy of PLD [100]. Another study using prostate cancer cell-derived exosomes promotes macrophage polarization into pro-tumorigenic M2 phenotype via AKT and STAT3 activation. Treatment with GW4869 significantly reduced exosome release and impaired M2 polarization. It also reduced macrophage-driven tumor promotion and suppressed prostate cancer progression in vivo [101].

GW4869 has also been shown to reduce CAF-derived exosome secretion by approximately 70% in vitro, both in untreated and gemcitabine-treated conditions. Treatment with GW4869 significantly decreased levels of Snail mRNA and miRNA-146a in Pancreatic ductal adenocarcinoma (PDAC) epithelial cells co-cultured with CAFs, indicating reduced transfer of chemoresistance-promoting signals. In co-culture experiments, blocking exosome release with GW4869 resulted in a significant reduction in epithelial cell survival during gemcitabine treatment (1 µM). In vivo, combining GW4869 with gemcitabine (100 mg/kg) in NOD/SCID mice implanted with AsPC1 and CAFs significantly inhibited tumor growth compared to gemcitabine alone, further demonstrating the role of GW4869 in disrupting exosome-mediated communication [102].

In severe acute pancreatitis, circulating vesicles rise markedly and contribute to intestinal barrier disruption. Administration of GW4869 significantly lowered their levels in affected rats, suppressing NLRP3 inflammasome-driven pyroptosis and mitigating intestinal injury, systemic inflammation, and secondary damage to organs such as the kidney and lung [103]. In another context, the same inhibitor was applied to pancreatic cancer models to explore the role of lncXIST in perineural invasion. Exposure of tumor cells to GW4869 (10 µM, 24 h) inhibited the transfer of this lncRNAto neural targets, resulting in reduced GDNF expression in SH-SY5Y cells and impairing the migratory influence of the tumor-derived secretome [104] (Fig. 4).

#### Ras-related proteins in the brain (Rab) inhibitors

Rab GTPases are key regulators of intracellular vesicle trafficking, including exosome biogenesis and secretion, and have been implicated in cancer progression, metastasis, and chemoresistance. Inhibition of specific Rab proteins such as Rab27a and Rab27b can disrupt MVB docking and fusion with the plasma membrane, thereby reducing exosome release. The silencing of Rab27A via siRNA decreases exosome secretion in multiple cancer models, disrupting MVB-plasma membrane docking and vesicle release [105]. Studies show that while GW4869 effectively inhibits ceramide-driven exosome production, it does not reduce Rab27A-dependent vesicle secretion, as observed in miR 23b experiments, where GW4869 failed to attenuate vesicle release when compared to Rab27A knockdown [106].

In a study, knockdown of RAB22A using siRNA reduced EGFR-positive microvesicles without significantly affecting classical exosome markers, such as CD63 or TSG101, indicating a distinct vesicle biogenesis pathway. Dominant-negative RAB22A-S19N mutant expression led to EGFR retention in early endosomes and reduced its plasma membrane recycling, suggesting Rab22A’s role in directing EGFR to outward-budding vesicles rather than lysosomal degradation. This trafficking route promotes the extracellular shedding of EGFR, contributing to paracrine oncogenic signalling in the tumor microenvironment [107]. In a study, a five-gene RAB prognostic model (RPM) including RAB10, RAB11A, RAB11B, RAB28, and RAB39B was constructed, and patients with a high-risk score (> 1) had significantly worse overall survival (AUC: 0.70–0.76). Drug-sensitivity analysis showed that high-risk groups were more responsive to erlotinib, lapatinib, and sorafenib, while low-risk groups responded better to axitinib, gefitinib, methotrexate, nilotinib, and sunitinib. The inhibition of RAB39B reduced sensitivity to ferroptosis inducers, such as RSL3 and erastin, suppressed LC3-II accumulation, and decreased malondialdehyde levels, indicating its role in autophagy-dependent ferroptosis [108, 109]. A combined use of GW4869 and Rab27A suppression yields an additive decrease in exosome-mediated signaling, indicating that they target non-redundant, complementary pathways in EVbiogenesis. This dual inhibition strategy may therefore provide broader suppression of tumor-derived vesicle communication [110].

#### Inhibiting exosome uptake in target cells

Inhibiting exosomes opens up another dimension in disrupting intercellular communication, which is crucial for tumour progression, metastasis, and therapy resistance. Approaches such as blocking heparan sulphate proteoglycans, inhibiting lipid raft-mediated endocytosis, or using pharmacological agents like Dynacare or amiloride effectively reduce exosome internalization. These interventions can attenuate exosome-mediated delivery of oncogenic cargo [99].

A recently published review has discussed how GW4869 and Rab inhibitors prevent exosome secretion by donor cells, but do not inhibit the uptake of exosomes by recipient cells. To block exosome uptake, strategies involving inhibitors of endocytosis, actin cytoskeleton modulators, or direct masking of exosome surface molecules (e.g., heparin or annexin-V) are typically employed. Exosomes released by cancer cells often carry oncogenic proteins, RNAs, and immunosuppressive signals that modulate the tumor microenvironment and instruct recipient cells to support malignancy. Blocking uptake pathways such as receptor-mediated endocytosis (e.g., using heparin), caveolin- or lipid raft-mediated internalization (e.g., using filipin III or methyl-β-cyclodextrin), or macropinocytosis (e.g., using amiloride) has been shown to reduce tumor cell invasiveness and stromal activation. These inhibitors prevent the functional delivery of exosomal cargo, thereby attenuating pro-tumorigenic signaling in recipient cells [111].

A study demonstrated that the inhibition of exosomes by heparin (10 µg/mL) reduced exosome uptake in MDA-MB-231 breast cancer cells, while amiloride (100 µM), a macropinocytosis inhibitor, achieved a 60% reduction. Inhibition of clathrin-mediated endocytosis using dynasore (80 µM) and of actin polymerization using cytochalasin D (10 µM) led to a 40–70% decrease in exosome internalization, depending on the cell line. These pharmacological inhibitors not only suppressed exosome uptake but also significantly downregulated downstream oncogenic signaling pathways, such as AKT and ERK phosphorylation. The study inferred that targeting uptake pathways provides an effective means to block exosome-mediated pro-tumorigenic effects [112].

While the above approaches aim to suppress oncogenic exosome-mediated communication by targeting their production, release, or uptake, an alternative therapeutic strategy exploits exosomes themselves as delivery vehicles for targeted nucleotide-based cancer therapies.

### Exosomes as therapeutic carriers

#### Antisense oligonucleotides

Antisense oligonucleotides (ASOs) bind to target RNAs through complementary base pairing and regulate gene expression post-transcriptionally. They exert their effect either by promoting RNA degradation via RNase H or by sterically blocking key regulatory processes like splicing or translation [113]. ASOs can modulate the expression of oncogenes either by directly targeting the mRNA in cells or by interfering with RNA species carried via exosomes, which are known to propagate oncogenic signals [114, 115]. Chemical modifications such as phosphorothioate backbones and 2′-O modifications improve ASO stability, cellular uptake, and target affinity, making them suitable for clinical applications [116]. A study on splice-switching ASOs to modulate the splicing of EGFR mRNA, a key oncogene in cancer progression and drug resistance. ASOs targeting EGFR exons 3, 18, and 21 successfully induced exon skipping in glioblastoma, liver, and breast cancer cell lines, with PNAT524, PNAT525, PNAT576, and PNAT578 as the most effective. The combined use of tyrosine kinase domain-targeting ASOs (PNAT576 + 578) enhanced transcript reduction and significantly suppressed cancer cell migration [117]. Another study, involving alternative splicing of PD-1 exon 3, which regulates the balance between full-length PD-1 (flPD-1) and soluble PD-1 (sPD-1), the latter of which can enhance anti-tumor immunity by blocking PD-1/PD-L1 interactions. Through mutational analysis, SpliceAid database mining, and pulldown assays, an exonic splicing enhancer (ESE) was identified, which is critical for exon 3 inclusion, and it was found that splicing factor SRSF3 binds this ESE to promote flPD-1 expression. A novel ASO was designed to disrupt SRSF3-ESE binding, resulting in increased exon 3 skipping and elevated sPD-1 expression [118].

The high specificity of ASOs allows for targeted treatment with minimal off-target effects compared to conventional therapies. It can also modulate the exosomal cargo, potentially altering tumor communication with the microenvironment. However, challenges such as poor stability in circulation, limited intracellular delivery, and the risk of immune activation or toxicity still limit their clinical application [119].

#### RNA interference

Endogenous nanovesicles, known as exosomes, can carry small interfering RNAs (siRNAs) to target cancer cells via membrane fusion, thereby bypassing the endosomal entrapment that often limits conventional RNA interference (RNAi) delivery methods [120, 121]. The engineered exosome, carrying siRNAs targeting RAD51, a gene critical for DNA repair and cancer cell survival, demonstrated that both chemically and electroporation-loaded exosomes could deliver functional siRNA into cancer cells (HeLa and HT1080), resulting in gene silencing, cell cycle arrest, and apoptotic cell death. Particularly, siRNA-loaded exosomes targeting RAD51 significantly suppressed cancer cell proliferation, suggesting the therapeutic potential of exosome-mediated RNAi [122]. In a study, biomimetic nanoparticles (CBSA/siS100A4@Exosome) utilize exosome membranes for lung-targeted delivery of siRNA to the pre-metastatic niche. These ~ 200 nm NPs demonstrated high biocompatibility, protected siRNA from degradation, and showed superior lung targeting and gene-silencing effects compared to liposome-based systems. In vivo results confirmed their potential to effectively suppress postoperative metastasis in triple-negative breast cancer (TNBC) [123]. Similarly, fibroblast-derived “iExosomes” carrying KRAS^G12D siRNA significantly inhibited pancreatic tumor growth in mouse models, signifying the feasibility of oncogene-specific RNAi therapy using exosomes [124].

Despite its potential strengths, several challenges exist that pose a hurdle to the application. For instance, native exosomes lack intrinsic target specificity and may enter non-malignant cells, raising concerns about toxicity; this issue is being addressed through RNA nanotechnology, where exosomes are decorated with tumor-specific ligands or aptamers, enhancing selective uptake and reducing off-target effects [121]. Moreover, exosome delivery also presents challenges such as endolysosomal degradation through cytosolic fusion, efficient production, consistent loading, and scalable manufacturing [125].

#### CRISPR/cas systems for RNA editing

Exosome-based delivery of CRISPR is another promising strategy for regulating gene expression through RNA editing in cancer therapy. Unlike DNA-targeting Cas9, RNA-targeting Cas13 variants (such as Cas13a, Cas13b/d) enable transient and reversible knockdown or base editing of oncogenic transcripts without permanent genome alteration [126, 127]. Fusion of catalytically inactive Cas13 with ADAR deaminase domains enables programmable A to I RNA base editing, suggesting precise correction of disease-linked RNA transcripts in cancer cells [126, 128]. To translate these RNA editors into therapy, exosomes derived from cancer or donor cells have been shown to encapsulate CRISPR/Cas9 plasmids, mRNAs, or RNP complexes and deliver them to recipient tumor cells with relative tropism and low immunogenicity [129, 130].

In a study, engineered exosomes loaded with CRISPR/Cas9 plasmids targeting the oncogenic KrasG12D mutation achieved efficient delivery of the Cas9-sgRNA complex into pancreatic cancer cells both in vitro and in vivo, leading to gene editing and suppression of KRAS signaling. The treatment significantly reduced tumor cell proliferation and inhibited tumor growth in mouse models without causing systemic toxicity. The exosomes showed excellent biocompatibility, avoided immune detection, and bypassed endosomal degradation to deliver their cargo efficiently [131] While Cas9 is known for editing DNA, Cas13-based platforms are designed to target and cleave RNA transcripts, making them ideal for transient silencing of oncogenic mRNAs such as KRAS, MYC, or BCL2 in tumors [132]. Unlike Cas9, which introduces permanent changes to the genome, Cas13 (especially Cas13a, Cas13b, and Cas13d variants) enables post-transcriptional gene silencing by degrading specific RNA sequences. This can be beneficial in cancer therapy, where reversible and precise control of oncogene expression is desirable without altering the genome. The Cas13 avoids potential off-target effects on DNA and can be delivered using exosomes or lipid nanoparticles for localized action in tumor cells [133].

#### Delivery mechanisms and loading strategies

Exosomes can be loaded with therapeutic cargo either endogenously (via genetic modification of donor cells) or exogenously using techniques such as electroporation, sonication, or incubation. Electroporation, for example, allows the encapsulation of siRNA or CRISPR/Cas components with high efficiency but may induce RNA aggregation if not optimized [122, 134]. Engineered exosomes are capable of evading endolysosomal degradation, thereby increasing intracellular delivery and therapeutic efficacy [135]. Engineered exosomes can deliver siRNA or shRNA to silence oncogenes, such as KRAS, MYC, or BCL2, thereby inhibiting tumor growth [118, 136, 137]. Additionally, exosomes transport tumor-suppressive miRNAs (e.g., miR-124) or inhibitors, such as anti-miR-21, to reprogram cancer cell behavior, and can deliver mRNA for cancer vaccines or immunotherapies [136]. Recent advances also include the exosome-based delivery of CRISPR/Cas systems for precise gene or RNA editing [138] and the encapsulation of small-molecule drugs (e.g., paclitaxel, doxorubicin) to enhance solubility and tumor-specific accumulation [139].

Exosome-mediated delivery systems have shown promising therapeutic potential across various cancer models. In pancreatic cancer, a study has demonstrated that MSCS-derived exosomes loaded with siRNA targeting KRASG12D significantly reduced tumor growth in a PDAC mouse model [140]. For TNBC Zhao et al. [123], developed a biomimetic nanoparticle system (CBSA/siS100A4@Exosome) by coating siRNA-loaded cationic bovine serum albumin with exosome membranes from autologous breast cancer cells, enabling targeted delivery to the lung pre-metastatic niche in TNBC. These ~ 200 nm nanoparticles showed superior lung affinity, protected siRNA from degradation, and effectively suppressed postoperative breast cancer metastasis in vivo [123]. Exosome-based delivery systems are advantageous over synthetic carriers, such as liposomes, due to their natural origin, which reduces the risk of immune system activation. Their small size and membrane composition enable better tissue penetration and cellular uptake, especially in tumor microenvironments. Apart from it, exosomes protect their cargo (e.g., RNA or proteins) from enzymatic degradation in the bloodstream, enhancing therapeutic stability. However, large-scale production of exosomes with consistent quality remains a technical hurdle. Other key challenges include inefficient cargo loading, lack of standardized purification methods, and limited progress in clinical translation and regulatory approval.

**Table 2:** A comparative overview of therapeutic strategies targeting exosome-mediated oncogenic communication is provided

Strategy	Targeted step	Approach type	Evidence stage	Key limitation	Reference
GW4869 (nSMase inhibitor)	Ceramide-dependent exosome biogenesis	Exosomes as therapeutic targets	In vitro, in vivo	Off-target effects; lack of pathway specificity	Trajkovic et al. [141]
Rab GTPase inhibitors (Rab27A/B, Rab22A)	MVB docking and exosome release	Exosomes as therapeutic targets	In vitro, in vivo	Limited selectivity; compensatory vesicle pathways	Ostrowski et al. [105]
Enhanced ORP9–ORP10 complex activity	PI4P clearance at ER–TGN contact sites limiting MVB formation and PM fusion	Genetic/protein gain-of-function	In vitro lipid-transfer and trafficking assays	PI4P depletion may broadly affect Golgi-dependent trafficking	He et al. [142]
Blockade of CD47–SIRPα signaling on exosomes	Circulating exosome persistence and immune evasion	Antibody-mediated neutralization	In vivo mouse models	Does not inhibit biogenesis; enhances immune clearance only	Kamerkar et al. [140], Zhao, [123]
Manumycin A	Post-transcriptional mRNA silencing via exosome suppression	Exosomes as therapeutic targets	In vitro	Off-target uptake; mechanism not exclusive to exosome pathways	Datta et al. [143]
Sulfisoxazole	DNA/RNA editing of oncogenes via exosome inhibition	Exosomes as therapeutic targets	In vitro, in vivo	Antibacterial activity; exosome inhibition not specific	Im et al. [144]
Inhibition of heparanase–syndecan–syntenin–ALIX pathway	Intraluminal budding and MVB-mediated exosome biogenesis	Genetic knockdown or enzymatic inhibition	Cell-based mechanistic studies	Affects specific exosome subpopulations rather than total output	Roucourt et al. [145]
Surfen (bis-2-methyl-4-amino-quinolyl-6-carbamide)	Heparan sulfate–dependent syndecan interactions upstream of ALIX-mediated budding	Small-molecule heparan sulfate antagonist	Cell-based biochemical assays	Indirect evidence for exosome inhibition; broad HS antagonism	Schuksz et al. [146]
LAMTOR1-derived peptides	PD-L1 sorting to lysosomes, reducing exosomal PD-L1 incorporation	Peptide-based modulation of trafficking	Preclinical (cell lines and murine models)	Stability and delivery challenges; safety not yet established	Wu et al. [147]
MFGE8-neutralizing antibodies	Sorting of PD-L1 into tumor-derived exosomes	Antibody-mediated blockade of MFGE8 signaling	In vivo preclinical models; patient correlations	Integrin pathway pleiotropy; delivery and safety require validation	Wang et al. [108, 109]

As summarized in Table 2, current therapeutic strategies targeting exosome-mediated oncogenic communication remain predominantly preclinical in nature, with most evidence derived from mechanistic cell-based studies and animal models. Pharmacological inhibition of exosome biogenesis, such as through neutral sphingomyelinase blockade using GW4869, has demonstrated effective suppression of tumor-derived exosome release and attenuation of pro-tumorigenic signaling in multiple cancer models; however, these effects are often accompanied by limited pathway specificity and off-target lipid signaling perturbations. Similarly, genetic or pharmacological interference with Rab GTPase–mediated MVB trafficking (e.g., inhibition of Rab27A/B or Rab22A) reduces exosome secretion but may activate compensatory vesicle transport pathways that partially restore EVoutput. More recently, targeting exosome cargo sorting and functional persistence, including modulation of PD-L1 incorporation into tumor-derived exosomes via LAMTOR1-derived peptides or MFGE8 neutralization, has revealed additional layers of immune-regulatory control. However, such approaches are constrained by delivery challenges and an incomplete understanding of exosome heterogeneity. Collectively, these examples illustrate that while individual strategies can disrupt specific steps of exosome biogenesis or function, none currently achieves comprehensive or selective inhibition of pathogenic exosome activity. These limitations underscore a significant translational gap and suggest that future therapeutic development will require improved mechanistic precision, integration of combination approaches targeting non-redundant exosome pathways, and systematic evaluation of safety and pharmacodynamic outcomes prior to clinical translation.

## Clinical progress and challenges

Recent years have seen significant strides in translating exosome-based strategies into oncology, particularly through their role in modulating oncogenes at the post-translational level. Several early-phase clinical trials have demonstrated the potential of engineered exosomes to deliver therapeutic cargo, such as small molecules, peptides, or RNA, that interfere with oncogenic protein modifications, stability, or activity. Exosomes, small EVs released by most cell types, have garnered growing interest due to their key role in cell communication and potential as therapeutic carriers [63]. Clinical trials are harnessing the unique properties of exosomes for targeted drug delivery in cancer therapy. For instance, a notable study involved MSC-derived exosomes carrying KRASG12D siRNA to treat metastatic PDAC [148]. Furthermore, small RNAs, such as siRNAs and miRNAs, are becoming central to these strategies by regulating gene expression and showing potential in cancer therapy [149]. While siRNA-based drug ONPATTRO has received FDA approval and others are under clinical evaluation, no miRNA drugs have yet been approved [150]. Hence, limitations, including low stability, immune responses, and poor cellular uptake, have prompted the development of optimized delivery platforms, such as lipid nanoparticles [148].

Exosomes are emerging as integral molecules due to their biocompatibility, safety, and uptake mechanisms, including phagocytosis, endocytosis, and fusion. These features make them effective carriers for therapeutic RNAs [63]. For instance, miR-134 delivered via exosomes suppressed breast cancer cell proliferation and enhanced sensitivity to anti-Hsp90 agents [151]. Similarly, engineered exosomes expressing GE11 or AS1411 and loaded with let-7a targeted EGFR on breast cancer cells, produce strong anti-tumor effects [152, 153]. A study by Kamerkar et al., also developed “iExosome,” an MSC-derived exosome loaded with KRASG12D siRNA that showed efficacy against pancreatic cancer [140]. Similarly, exosomes loaded with vascular endothelial growth factor siRNA have been shown to prevent brain tumor angiogenesis and exhibit anti-tumor effects [154]. Furthermore, the integration of CRISPR-Cas9 into exosome-based therapies is another emerging frontier. It was observed that exosomes from SKOV3 cells, when electroporated with CRISPR-Cas9 targeting PARP-1, led to reduced tumor growth [129, 155, 156]. Other strategies involve the use of hybrid exosome-liposome carriers for improved encapsulation and delivery of large nucleic acids [157]. Additionally, the surface engineering of exosomes has expanded their therapeutic applications to treat various diseases. For example, exosomes presenting signal-regulatory protein alpha (SIRPα) or hyaluronidase PH20 have been shown to enhance anti-tumor immune responses [158, 159]. Moreover, epigenetic modifications are now recognized as crucial for fine-tuning the response of cancer cells to various therapies, and the development of acquired resistance against targeted therapies often involves dysregulated epigenetic modifications. Depending on the cargo’s constitution, exosomes can influence several epigenetic events, thereby impacting post-translational regulations. Hence, the role of exosomes as facilitators of epigenetic modifications has come under increased scrutiny in recent years. Exosomes can deliver methyltransferases to recipient cells, which can affect the expression of several oncogenes and tumor suppressors, thereby impacting cancer therapy resistance [160]. For instance, a study focusing on fibroblasts showed that the exosome-mediated transfer of miR-29b from CAFs to HCC cells resulted in the miR-29b-mediated suppression of DNA methyltransferase 3b in the HCC cells [161]. Thus, exosomes can carry cargo to affect methyltransferases in the recipient cells with effects on their cellular phenotype [160]. Together, these clinical and preclinical advances underscore the therapeutic potential of exosomes in regulating oncogenes post-translationally. Nonetheless, challenges remain in standardization, which must be addressed to fully harness their clinical utility.

Despite the promising therapeutic potential of exosomes in cancer treatment, several hurdles hinder their clinical translation, demanding further research and innovation. One major challenge lies in large-scale production with consistent quality [118]. Exosomes are complex biological vesicles secreted via intricate cellular mechanisms, making their manufacturing demanding and costly. Moreover, different therapeutic applications may necessitate tailored modifications, further complicating the manufacturing process and elevating costs. Therefore, cost-effective production techniques are crucial for facilitating the widespread clinical application of exosome-based therapies [162]. Another significant limitation is the lack of a clear regulatory framework. In cancers such as liver and colorectal, this regulatory ambiguity may delay clinical progress [163]. Additionally, exosomes derived from tumor cells have been shown to carry oncogenic molecules that can disrupt normal cellular function, potentially promoting tumor progression and metastasis [164]. Thus, comprehensive preclinical evaluation is essential to mitigate such risks and ensure patient safety. Clinical translation of exosome therapies remains in its early stages. Although preclinical studies have produced encouraging results, a few clinical trials have also progressed to advanced phases. In cancers like prostate and lung, current trials are still preliminary, limiting conclusions regarding efficacy and safety [118, 165]. Also, long-term effects and potential adverse outcomes need thorough investigation before exosomes can be incorporated into routine cancer therapy. Thus, addressing these gaps will require robust clinical studies that focus on both therapeutic efficacy and long-term safety [81, 82].

Despite compelling preclinical findings, one must recognize the numerous limitations of current experimental models. The majority of functional studies rely on in vitro cell culture techniques or xenograft mouse models, which may not accurately reflect the patients’ tumor heterogeneity, immunological interactions, and microenvironmental complexity [3, 166]. Furthermore, exosomal RNA’s functional effects and composition might vary significantly depending on the type of tumor, which limits its generalizability to other cancers. Experimental heterogeneity resulting from different characterisation techniques, RNA quantification techniques, and separation procedures (ultracentrifugation, precipitation, size-exclusion chromatography) makes cross-study comparability and repeatability more difficult [166]. These characteristics present potential translational issues, including inconsistent dose, off-target effects, and ambiguous biodistribution in clinical settings. Moreover, practical limitations—including low exosome yields from biofluids, the high costs and labor associated with large-scale isolation, and challenges in ensuring consistent purity and quality at scale—impede clinical translation. The lack of established regulatory frameworks and ongoing safety uncertainties, including potential immunogenicity and oncogenic cargo, complicates the advancement of exosome-based therapeutics. In order to reconcile experimental results with therapeutic implementation, these variables underscore the need for standardized techniques, comprehensive preclinical assessment, and well-planned clinical trials [81, 82, 167].

## Future directions

Recent advances in exosome research highlight their vast potential in diagnosis, therapy, and prevention across various diseases. In diagnostics, exosomes act as non-invasive biomarkers for early detection and monitoring of cancer, cardiovascular, and neurodegenerative diseases through liquid biopsy approaches [118, 168]. Therapeutically, exosomes are being explored as natural drug delivery vehicles owing to their biocompatibility, low immunogenicity, and ability to cross biological barriers. Studies have shown that exosomes are efficient in delivering small molecules, RNA therapeutics, and anti-cancer agents with targeted tissue delivery and minimal off-target effects [169]. Additionally, exosomes have shown promising results in immunomodulation, including vaccine development through exosome-based antigen delivery. Therefore, in the future, exosomes have the potential to revolutionize cancer therapy, as these molecules can be used in personalized therapy using patient-derived exosomes to reduce immune rejection and deliver precision therapeutics, such as CRISPR-Cas9, especially for hard-to-treat cancers like glioblastoma [81, 82, 170]. These may also support immunotherapy by transporting checkpoint inhibitors or tumor antigens, and modulate the tumor microenvironment to inhibit angiogenesis or enhance immune infiltration [81, 82]. Advances in engineering exosomes for CRISPR-Cas9 or siRNA delivery hold promise, although further improvements in loading and targeting are still needed [171]. Additionally, the integration of hybrid exosomes and AI in biomarker discovery and therapeutic simulation may further accelerate the clinical translation of these technologies. By addressing these questions, researchers can unlock the full potential of exosomes as diagnostic and therapeutic tools. These future directions aim to bridge the gap between innovation and implementation, advancing the field toward transformative clinical impact [171].

## Conclusion

Despite remarkable progress, critical clinical challenges remain in translating exosome-based technologies into mainstream oncology. A major obstacle lies in the inherent heterogeneity of exosomes, which complicates the production of consistent, reproducible therapeutics. Standardized and scalable methods for exosome isolation, purification, and characterization are urgently needed to ensure quality control across clinical applications. Moreover, determining the optimal cell sources for therapeutic exosome production and improving cargo loading efficiency without compromising vesicle integrity remains an unresolved issue. Furthermore, exosomes hold transformative potential in precision oncology; thus, the ability to function as minimally invasive biomarkers, targeted drug delivery systems, and immunomodulators positions these particles as next-generation tools in cancer care. Additionally, the integration of artificial intelligence for biomarker discovery, exosome engineering for gene and RNA delivery, and ongoing clinical trials indicate a promising path towards cancer therapy. Moreover, personalized exosome-based therapeutics may one day overcome the tumor heterogeneity and resistance mechanisms that currently limit effective treatments. As technological and regulatory hurdles are addressed and clinical pipelines mature, exosomes are poised to redefine the landscape of cancer therapy, offering safer, more targeted, and patient-specific solutions that could significantly improve outcomes on a global scale.

## Data Availability

No datasets were generated or analysed during the current study.
